# International Congress on Transposable elements (ICTE 2016) in Saint Malo: mobile elements under the sun of Brittany

**DOI:** 10.1186/s13100-016-0075-7

**Published:** 2016-10-20

**Authors:** Pascale Lesage, Mireille Bétermier, Antoine Bridier-Nahmias, Michael Chandler, Séverine Chambeyron, Gael Cristofari, Nicolas Gilbert, Hadi Quesneville, Chantal Vaury, Jean-Nicolas Volff

**Affiliations:** 1 Paris Diderot, Sorbonne Paris Cité, INSERM U944, CNRS UMR 7212, Institut Universitaire d’Hématologie, Hôpital Saint Louis, 75010 Paris, France; 2 Institute of Integrative Biology of the Cell (I2BC), CEA, CNRS, Univ Paris-Sud, Université Paris-Saclay, 91198 Gif-sur-Yvette, France; 3 Department CASER Conservatoire national des arts et métiers (Cnam), 75003 Paris, France; 4 Laboratoire de Microbiologie et Génétique Moléculaires, Centre National de Recherche Scientifique, Unité Mixte de Recherche 5100, 118 Rte de Narbonne, 31062 Toulouse Cedex, France; 5 Institut de Génétique Humaine, CNRS, UPR1142, 34396 Montpellier Cedex 5, France; 6 Institute for Research on Cancer and Aging in Nice (IRCAN), CNRS UMR 7284, Inserm U1081, Faculty of Medicine, University of Nice-Sophia Antipolis, Nice, France; 7 Institute for Regenerative Medicine and Biotherapy, INSERM, U1183 Montpellier, France; 8 INRA, UR 1164, URGI, Unité de Recherche en Génomique-Info, 78026 Versailles Cedex, France; 9 Laboratoire GReD, Université Clermont Auvergne, CNRS, Inserm, BP 10448, 63000 Clermont-Ferrand, France; 10 Institut de Génomique Fonctionnelle de Lyon, Ecole Normale Supérieure de Lyon, 69364 Lyon Cedex 07, France

**Keywords:** Transposable elements, Evolution of transposable elements, Impact on genomes, Control of transposition, Mechanism of transposition, Transposon-based gene therapy

## Abstract

The third international conference on Transposable Elements (ICTE) was held 16–19 April 2016 in Saint Malo, France. Organized by the French Transposition Community (Research group of the CNRS: “Mobile genetic elements: from mechanism to populations, an integrative approach”) and the French Society of Genetics, the conference’s goal was to bring together researchers who study transposition in diverse organisms, using multiple experimental approaches. The meeting gathered 180 participants from all around the world. Most of them contributed through poster presentations, invited talks and short talks selected from poster abstracts. The talks were organized into six scientific sessions: “Taming mobile DNA: self and non-self recognition”; “Trans-generational inheritance”; “Mobile DNA genome structure and organization, from molecular mechanisms to applications”; “Remembrance of (retro)transposon past: mobile DNA in genome evolution”; and finally “The yin and the yang of mobile DNA in human health”.

## Introduction

Transposable (or mobile) genetic elements play a crucial role in the living world and are present in all domains of life. These jumping genes, originally thought to be a genetic curiosity, are proving to be ubiquitous and immensely important. They have a profound impact on their host genomes in whatever organism they occupy, through their ability to drive DNA rearrangements, generate mutant alleles, activate gene expression and sequester and transport a large variety of genes. In bacteria, this includes antibiotic resistance, symbiosis and pathogenicity factors. In mammals, they are implicated as the cause of many genetic diseases and are involved in various cancers both as drivers and as cancer markers. They are equally important in the plant world (where their effects were first recognized); they are involved in genome modifications resulting in modifications of fruiting quality, anthocyanin (antioxidant) and plant variegation. Many transposable elements are no longer mobile and now perform essential host functions, such as immunoglobulin switching or telomere maintenance. The study of these genetic elements has also provided clues on some of the most unusual regulatory mechanisms, the most notable being translational and transcriptional frame-shifting, regulation by RNAi and other RNA signaling pathways and epigenetic control.

The goal of ICTE 2016 was to provide a multidisciplinary forum through which scientists from a variety of generally non-overlapping scientific areas can meet and take advantage of each other’s expertise. In the past meetings, this has proved to be a fertile hunting ground. In addition to exchanges of ideas, this meeting also serves to increase awareness of the power of new cutting-edge technologies in DNA and RNA sequencing, in genetics, genomics, biochemistry and bioinformatics.

## Keynote lectures

Eukaryotes contain small regulatory RNAs that have been referred to as the dark matter of genetics. They are associated with Argonaute proteins. Some of these small RNAs guide the Argonaute proteins to a complementary RNA and act as negative regulators of gene expression either at the level of transcription, messenger RNA turnover or translation. Other small RNAs participate in more complex epigenetic systems affecting chromatin or act as part of an RNA signal that moves between cells. In his keynote lecture, Sir **David Baulcombe** (Department of Plant Sciences, University of Cambridge, UK) addressed the following question: to what extent does epigenetics contribute to natural variation? Can it explain transgressive phenotypes, when a plant progeny shows different phenotypes than any of the parents? He reported that during hybridization in plants by crossing different tomato varieties 24-nucleotide small RNAs (sRNAs) target new mRNAs and produce secondary sRNAs that are specific to a given hybrid. These sRNAs are transported from shoots to roots. By grafting Arabidopsis wild type shoots on a root system deficient for sRNA production, his group showed that LINEs are targeted to silence as well as, and unexpectedly, loci with partial complementarities. These findings lead to a revised view of how transposons and repeated sequences might influence the establishment of new heritable variations. Sir David also described mutants affecting RNA-Directed DNA methylation (RdDM) in tomatoes. Their mutant phenotypes will provide important insights into the evolutionary history of the RdDM pathway and its function.

Another highlight of the congress was the keynote lecture by **Emmanuelle Charpentier** (Max Planck Institute for Infection Biology, Department of Regulation in Infection Biology, Berlin, Germany) who recalled the history of the CRISPR-Cas9 system’s discovery. Since the first recombinant DNA molecule produced in the 70’s, much progress has been made towards easy genome editing but no discovery other than CRISPR-Cas9 has received such a critical acclaim and such an echo in the public mind. Its biological raison d’être is as an RNA-mediated adaptive immune mechanism that protects prokaryotes from exogenous DNA. CRISPR are regions of prokaryotic genomes containing short repetitions separated by spacer sequences, which are homologous to foreign elements such as plasmids or phages. After transcription and maturation, the resulting RNA species are able to guide the Cas9 endonuclease to cleave target DNA and protect the cell from the invasion. This system offers new opportunities for precise genome modification and has the advantage of being easy to use, able to simultaneously correct multiple sites in the genome and also less expensive than other nucleases. Successful genome editing and silencing based on CRIPSR-Cas9 has been reported in many species including yeast, plant, mouse and human cells. In her lecture, E. Charpentier described the biological roles of CRISPR-Cas9, the mechanisms involved, the evolution of type II CRISPR-Cas components in bacteria and the applications of CRISPR-Cas9 as a novel genome engineering technology.

## Session 1: taming mobile DNA activity: self and non-self recognition

Transposable elements (TEs) are silenced by small germline RNAs called Piwi-interacting RNAs (piRNAs). Long single-stranded transcripts are precursors for piRNA biogenesis, but how transcripts are recognized as precursors was not known. **Ramesh Pillai** (University of Geneva, Switzerland) reported that a sequence element called piRNA-trigger sequence (PTS) at the 5′ end of the Drosophila piRNA cluster transcript flamenco is sufficient to recruit any transcript into the piRNA biogenesis pathway. Processing proceeds in a 5′-to-3′ direction, converting sequences downstream into ~26-nucleotides piRNAs, and this proceeds for kilobases and even through structured regions like tRNA sequences. A second mechanism to identify transcripts as precursors is their cleavage by a cytosolic Piwi protein in the fly and mouse germlines. Piwi slicing launches waves of non-overlapping piRNA generation in the 5′–3′ direction. Such piRNAs are preferentially loaded onto fly and mouse nuclear Piwi proteins. This process serves to communicate post-transcriptional RNA slicing by cytosolic Piwi proteins to the transcriptional repression machinery represented by nuclear Piwi proteins. An inchworm model was presented in which the movement of the processing machinery along the transcript cleaves and simultaneously generates 5′ and 3′ ends of new primary piRNAs.

TEs probably account for more than two-thirds of the human genome, and as genomic threats they are subjected to epigenetic control imposed from the earliest stages of embryonic development. In his presentation, **Didier Trono** (EPFL, Lausanne, Switzerland) described how TE sequences provide a high-density barcode for cell identity, activation state and differentiation status. He showed that an important component of this process is the sequence-specific recognition of TEs by KRAB-containing zinc finger proteins (KRAB-ZFPs), a large family of transcription factors that act by recruiting inducers of heterochromatin formation and DNA methylation via their cofactor TRIM28. In light of these data, D. Trono concluded that transposons and their KRAB-ZFP controllers contribute together to the establishment of transcriptional networks that likely influence all aspects of human biology.

Recent studies have revealed a critical role of the piRNA pathway in controlling L1 expression. Indeed, loss-of-function mutations in many piRNA pathway genes manifest a severe defect in fetal piRNA biogenesis (predominantly those derived from retrotransposons), elevated L1 RNA and protein levels, and an ultimate meiotic failure phenotype. However, technical difficulties have precluded quantitative analyses of L1 retrotransposition and its role in meiotic progression. **Wenfeng An** (South Dakota State University, Brookings, USA) developed a transgenic mouse model, in which an L1 transgene is regulated by an endogenous mouse L1 promoter. Sporadic retrotransposition was observed in the wild-type background. A greater than 300-fold increase in retrotransposition was detected specifically in meiotic germ cells of piRNA pathway knockout mice. This work has important implications on the developmental timing of L1 retrotransposition and the functional impact of L1-mediated genomic instability in piRNA-deficient genetic backgrounds.

## Session 2: from molecular mechanisms to applications

The session on transposition mechanisms included talks which addressed cellular control of retrovirus integration, Alu RNP crystal structure, integron evolution, the biochemical properties of Sleeping Beauty and helitron transposon systems and their use for gene transfer in mammalian cells, and the development of new methods to identify actively transposing element families in any eukaryotic genome.

**Fred Dyda** (NIDDK, NIH, Bethesda, USA) started the session by describing a class of transposases, HUH or Y1 transposases. These use single strand DNA as substrates and an active site tyrosine, which forms transitory 5′ phospho-tyrosine enzyme-DNA intermediates in which the histidine-hydrophobic amino acid-histidine triad is used in coordinating an essential divalent metal cation. The major advances presented focused on recent work on helitrons, eukaryotic transposons, which probably transpose using a rolling-circle mechanism, like the prokaryotic IS*91* insertion sequence family. In collaboration with the Ivics group, they reconstructed an ancient element from a bat genome, which they call hellraiser. They showed that hellraiser is active in human cells. It was also shown that hellraiser generates closed-circular DNA molecules as probable intermediates and that a hairpin structure at the hellraiser 3′end is important for this.

Integration site selection is a key feature of host-mobile DNA coevolution. **Paul Lesbats** (Clare Hall Laboratories, London, UK) presented data supporting a role for the viral structural protein Gag in directing the integration of the prototype foamy virus (PFV). Biochemical and structural studies indicate that PFV GAG directly binds to an acidic patch at the surface of nucleosome octamers, through a conserved and arginine-rich C-terminal domain, named the chromatin binding sequence. Altering this interaction redirects Gag and viral integration toward centrosome and centromeric regions, respectively. Future Gag ChIP-seq studies should provide additional insights on this process.

Integration is not the only the fate of transposable element DNA. **Marie Mirouze** (IRD, Montpellier, France) took advantage of TE extra-chromosomal DNA forms, a possible by-product of TE mobilization, to identify actively transposing TE families among all copies, through DNA circle purification and sequencing. This original approach, named mobilome-seq, allowed successfull identification of all known active TEs in Arabidopsis or rice, and could be applied to many plant and animal species to screen for TE mobilization under various physiological or stress conditions, including in very large and repetitive genomes.

How non-autonomous retrotransposons hijack the replicative machinery of other retroelements is not well understood. **Oliver Weichenrieder** (Max Planck Institute, Tübingen, Germany) has explored this phenomenon by obtaining the crystal structure of a minimal and active Alu ribonucleoprotein particle (RNP). Alu elements can use LINE-1 ORF2p, a protein with endonuclease and reverse transcriptase activities, for their mobilization. Interestingly, the Alu RNP structure suggests recognition of stalled ribosomes that appear during ORF2p translation, and suggests a co-translational mechanism for recruiting the retrotransposition machinery into Alu RNP complexes.

The talk given by **Zoltán Ivics** (Paul Ehrlich Institute, Langen, Germany) nicely illustrated various approaches to gene therapy in mammals using a number of different transposon derivatives many of which were tagged with fluorescent markers, which permitted their visualization in the animal. The presentation gave an overview of present advances in the field. He showed that the Sleeping Beauty transposon can efficiently establish stable gene transfer after delivery into the germline of several mammalian species, including mice, rats, rabbits, pigs and cattle, that insertion occurs in a quasi-random fashion in human T cells (with the implication that it might be safer than other gene delivery systems). Finally the crystal structure of the Sleeping Beauty transposase is now being used as a guide for generating “designer” next-generation transposases.

The final presentation of this session by **Didier Mazel** (Pasteur Institute, Paris, France) concerned the IntI integron integrase, a tyrosine recombinase that catalyzes acquisition of integron cassettes. Int proteins have the unique property among all tyrosine-recombinases to recombine folded single strand DNA substrate. This is the result of an additional domain, which inhibits activity on the complementary strand. He presented evidence that in spite of its preference for its single strand DNA substrate, IntI exhibits an inefficient activity for double strand DNA substrates. This suggests that IntI may have evolved from canonical recombinases having retained some activity on double strand DNA substrates while evolving its present activity in cleaving its folded, single strand DNA substrate.

## Session 3: remembrance of (retro)transposon past: mobile DNA in genome evolution

**Clément Gilbert** (CNRS, Poitiers, France) studied gene exchange between viruses and their hosts using the AcMNPV baculovirus, which infects caterpillars of the moths *Trichoplusia ni* and *Spodoptera exigua.* He observed a large diversity of moth sequences transferred into viral genomes after infection, including DNA transposons that integrate through a canonical reaction of transposition, but also other sequences inserted via microhomology-mediated recombination. About 5 % of viruses in a given AcMNPV population carry at least one unfixed moth sequence (mostly TEs), further supporting the role of viruses as vectors of horizontal transfer of genetic material between their hosts.

A very convincing demonstration of TE-mediated regulatory network rewiring was presented by **Cédric Feschotte** (University of Utah, Salt Lake City, USA), who described how Endogenous RetroViruses (ERVs) have dispersed multiple interferon (IFN)-inducible enhancers independently in diverse mammalian genomes. In particular, many ERV-derived enhancers have been retained near innate immunity genes. The functional importance of some of these HERV-derived sequences was demonstrated in vivo by CRISPR-Cas9 deletion experiments in human cells. This revealed their involvement in the regulation of essential immune functions including the AIM2 “inflammasome”. In conclusion, ERV-derived IFN enhancers have probably been coopted repeatedly during mammalian evolution, fueling lineage-specific genetic innovation in immune responses.

For digging up the past, **Florian Maumus** (INRA, Versailles, France) has established methods for deep repeat annotation in genomes, allowing detection of ancestral repeated sequences. His study provided new insights into the evolutionary dynamics of genomes and chromosomes, as exemplified by the detection of a remnant paleocentromere vanishing as the result of a chromosome fusion in chromosome 1 of *A. thaliana*. The paleocentromeric region presented several peculiarities compared to other regions on chromosome arms. In particular, it is associated with increased deletion and recombination rates, suggesting an important hotspot for allelic diversity.

**David Pollock** (University of Colorado School of Medicine, Denver, USA) presented *AnTE*, software implementing a Bayesian method to predict the ancestral sequence of highly duplicated TEs. This eliminates major problems encountered with the consensus sequence approaches. Remarkably, it improves the analysis of substitution rates and replication timing, introduces simultaneous alignment, distribution of replication ages, and allows the analysis of one million sequences simultaneously. The analysis of ~800 k Alu sequences showed that replicating sequences are highly constrained, and that the identity of functional sites fluctuates over time. Inference of replication time indicated that ancestral replicating sequences exhibit a much narrower range of activity than previously realized. A much more accurate estimation of timing suggested the coevolution of Alu with LINEs and/or the primate host. According to D. Pollock’s results, the use of consensus sequences and single phylogenetic trees should be abandoned in favor of ancestral sequences and phylogenetic networks.

To better understand the roles of TEs in the adaptation of their host, **Josefa Gonzalez Perez** (Institut de Biologia Evolutiva, Barcelona, Spain) identified a set of candidate adaptive insertions through the analysis of the frequencies of 1,495 euchromatic TEs in four natural populations of *Drosophila melanogaster*. Strikingly, most insertions were located in gene promoters or in the first intron, suggesting roles in gene regulation. Many candidate TEs were located nearby genes involved in stress response, suggesting roles in adaptation to environmental changes. Remarkably, one insertion was located in an evolutionary hotspot and was associated with increased tolerance to cold stress.

## Session 4: the Yin and the Yang of mobile DNA in human health

As we all know, mobile elements are a mutational force in all genomes. In humans, the initial demonstration of LINE-1 activity was the characterization of an insertion in the factor VIII gene resulting in hemophilia (Kazazian et al. 1988, *Nature* ***332***). In her presentation, **Kathleen Burns** (Johns Hopkins University School of Medicine, Baltimore, USA) described how mobile elements of our genome contribute to heritable structural variation. Although the majority of insertions have no phenotypic effect, genome-wide association studies suggested potential exception in insertions that occur at disease-risk alleles. She also described her recent studies on acquired LINE-1 insertions and LINE-1-encoded protein ORF1p expression in several cancer types.

As recalled by **Goeffrey Faulkner** (University of Queensland, Brisbane, Australia) in his talk, LINE-1 mobilization in brain is well established. However, of the frequency of L1 transposition remains unknown. Current estimations range from one insertion/300 cells to 10 insertions/cell. Here, G. Faulkner presented his recent work on LINE-1 mobilization during mammalian development. His laboratory developed a mouse retrotransposon capture sequencing (RC-seq) protocol and applied it to a pedigree of ~100 mice. They observed approximately one new heritable LINE-1 per 8 live-born mouse offspring. They also showed that the majority of heritable LINE-1 insertions arose in the early embryo, though some also likely occurred in germ cells and could potentially be transmitted to the next generation.

Similarly, **Jose-Luis Garcia-Perez** (Centro Pfizer-Universidad de Granada, Spain) is interested in LINE-1 insertion during human embryogenesis, studying in particular the pattern of LINE-1 expression and endogenous retrotransposition during early embryogenesis. He described his work deciphering the timing and rate of heritable retrotransposition using cellular models and other approaches.

**Alex Bortvin** (Carnegie Institution for Science, Baltimore, USA) outlined key lessons that his group has learned by studying mutually antagonistic relationship between germ cells of mice and retrotransposon LINE-1. Their data suggest the critical role of a balance between the extent of epigenetic reprogramming during cell differentiation and the activity of transposon-restraining mechanisms. Failure to silence reactivated L1 elements results in the accumulation of at least two distinct triggers of cell death (DNA damage and L1 RNA:DNA hybrids) and preferential elimination of L1-overexpressing cells from developing tissues. Based on these data, A. Bortvin proposed that L1 elements might positively contribute to normal development by pruning cells whose epigenetic remodeling exceeds their restraining capacity. He further speculated that L1 overexpression in cells deficient in mechanisms sensing/responding to L1-generated triggers such as checkpoints or innate immunity could lead to accumulation of tumorous cells with elevated L1 expression and new L1 insertions.

**Marie-Elisa Pinson** (GreD, CNRS, INSERM, Clermont-Ferrand, France) is studying the impact of LINE-1 hypomethylation observed in cancer and particularly in gliomas. This chromatin status not only favors LINE-1 transcription, but also allows the formation of LINE-1 chimeric transcripts (LCTs) generated from the LINE-1 antisense promoter. Based on a dedicated bioinformatics tool, CLIFinder, her laboratory identified in the genome of 13 gliomas and 3 controls brains a total of 3000 chimeras composed of LINE-1 5′UTR followed by a unique adjacent sequence. Most but not all of these chimeric transcripts have been found in tumor samples, and a majority is implying young full length LINE-1. Remain to be established if LCTs have a functional role in the tumorigenic process.

The next presentation focused on the regulation of LINE-1 elements. It is known, from the study of many animal models, that small RNAs, like piRNAs, siRNAs or miRNAs, are largely implicated in the control of mobile elements. Dicer protein is one of the key partners implicated in the pathway generating such small RNAs. **Constance Ciaudo** (ETH, Zurich, Switzerland) generated a new Dicer knockout in mouse embryonic stem cells using CRISPR/Cas9 system. She observed new stem cell phenotypes associated with a strong up-regulation of active LINE-1 elements. This up-regulation is present at the protein level but leads to a weak increase in retrotransposition events. She is currently investigating the impact of LINE-1 deregulation on stem cells differentiation capabilities.

The last presentation of the session was from **Erez Levanon** (Bar-Ilan University, Ramat-Gan, Israel). His work evaluates the impact of APOBEC proteins not only on the restriction of LTR-retrotransposons activity but also on their potential for increasing genomic sequence diversity. APOBECs defend the genome against viruses and LTR-retrotransposons by C-to-U DNA editing. E. Levanon convincingly showed that edited retrotransposons, which become defective upon insertion, could be preferentially retained in active genomic regions. In this way, these copies could accelerate genome evolution by enhancing the probability of their exaptation for novel function.

## Session 5: transgenerational inheritance

The session on transgenerational inheritance included three talks that described the role of small non-coding RNAs in transposon repression, programmed elimination of germline transposon-related genomic sequences during somatic differentiation, or resistance against viral infection.

**Eric Miska** (Gurdon Institute, Univ. of Cambridge, UK) discussed the biology of siRNAs and piRNAs in the nematode *C. elegans*. In particular, he showed how the interaction of these non-coding RNA pathways with both the unique and repetitive parts of the genome leads to striking heritable effects independent of genomic DNA sequence. He also provided direct evidence that environmental exposures in one generation (in this case odourants) can affect *C. elegans* behavior lasting for 2–3 generations. Finally, he stressed how small RNA pathways, as part of the defense system against viruses and transposable elements, evolve rapidly, even within the nematode phylum, and that this must be considered when trying to generalize observations from a single species.

**Eric Meyer** (IBENS, Ecole Normale Supérieure, Paris, France) reported novel observations that challenge the current model for RNA-mediated epigenetic control of programmed germline transposon-related sequence Internal Eliminated Sequence (IES) elimination, during somatic macronucleus development in the ciliate *Paramecium tetraurelia*. According to the model, short non-coding scnRNAs are initially produced from the entire germline genome during meiosis and titrated against the maternal macronuclear genome. Titration results in selection of germline-specific scnRNAs that will target excision of their homologous sequences in the developing zygotic macronucleus. The scnRNA pathway is consistent with the observed maternal inheritance of mating types after conjugation. This depends on scnRNA-targeted excision of a co-opted IES spanning the promoter of a mating-type determination gene. A prediction of the model is that a divergent IES, introduced into the zygotic genome (by conjugation between allelic strains) would not be excised properly in the F1 exconjugant issued from the parent that did not already harbor the IES in its germline genome, because no homologous maternal scnRNAs would have been produced during meiosis. Experiments from the Meyer laboratory contradict this prediction. They demonstrate that paternal scnRNAs, dependent upon the presence of paternal Piwi proteins, can efficiently program excision of divergent allelic IESs. The exact mechanism remains unknown (transmission of paternal scnRNAs through cytoplasmic exchange, or genomic imprinting in paternal gametic nuclei before fertilization).

**Mark Kunitomi** (laboratory of Raul Andino, UCSF, San Francisco, California) presented evidence that the piRNA pathway, via Piwi4, plays a role in somatic antiviral immunity in the mosquito *Aedes aegypti*. He reported that production of piRNA from virus sequences depends on endogenous reverse transcriptases that synthesized DNA from infecting viral RNA. In addition, the *A. aegypti* genome contains a plethora of sequences with similarity to non-retroviral RNA viruses, called Endogenous Viral Elements (EVEs). M. Kunitomi presented evidence that host EVEs act as piRNA-producing loci that may contribute to anti-viral resistance.

## Session 6: mobile DNA in genome structure and organization

**Henry Levin** (NIH, Bethesda, USA) provided another nice example of gene network rewiring by TEs. Deep sequencing of integration sites in diploid cells shows that the Tf1 retrotransposon in *Schizosaccharomyces pombe* preferentially integrates into promoter sequences of stress response genes and increases their expression. To assess whether Tf1 was able to rewire stress response genes, yeast strains with 40,000 insertions were grown with cobalt chloride, mimicking hypoxia, and deep sequencing revealed that integration in a specific set of genes allowed certain cells to become a larger fraction of the culture. The TORC1/2 kinase pathway seems to be involved, and genetic experiments demonstrated that a single insertion next to genes resulted in resistance to cobalt by altering TORC expression levels. In another study, his laboratory generated an ultra dense map of HIV-1 insertions in HEK293T cells and revealed that integration occurred more frequently in highly spliced genes. Affinity purification experiments showed that LEDGF/p75, already well known to be responsible for HIV targeting to actively transcribed genes, interacts with multiple splicing factors. The group proposes that interactions with splicing factors tethers the integrase to highly spliced genes and results in integration in intronic sequences.

**Vivien Measday** (University of British Columbia, Vancouver, Canada) has investigated the role of the nuclear pore complex (NPC) in yeast Ty1 retrotransposition. She presented data indicating that Ty1 mobility decreases in mutants of the nuclear pore inner ring and nuclear basket complexes but increases in a *nup120* outer ring mutant. NPC mutants affect Ty1 mobility at different steps of Ty1 replication cycle but do not affect the nuclear localization of Ty1 integrase. Some NPC mutants with wild-type levels of Ty1 mobility presented some alterations in the pattern of Ty1 integration upstream of Pol III-transcribed genes, suggesting that Ty1 could be mis-targeted in these mutants.

**Antoine Bridier-Nahmias** (IUH, Inserm, CNRS, Paris Diderot University, France) reported that the AC40 subunit of RNA Pol III, which interacts with Ty1 integrase (IN), is the predominant determinant targeting Ty1 upstream of Pol III transcribed genes. A six amino-acid domain at the IN C-terminus is necessary and sufficient for interaction with AC40 and Ty1 targeting to Pol III-transcribed genes. The association of Ty1 insertion sites with specific chromatin features in the absence of AC40/IN interactions suggests a secondary target site preference of Ty1. When Ty1 integrates into coding genes, it favors poorly transcribed genes.

**Irina Arkhipova** (Marine Biological Laboratory, Woods Hole, USA) mentioned that genomes of bdelloid rotifers harbor giant retrotransposable elements, Terminons, of up to 40 kb unit length. These elements can attach to exposed G-rich overhangs at telomeres via short stretches of telomeric repeats, and can form long head-to-tail chains extending up to 100 kb. The principal polymerizing components are intron-containing reverse transcriptases from the PLE (Penelope-like elements) class. Terminons contain multiple co-oriented ORFs, which code for a variety of enzymatic and structural functions and their replication follows the master copy model, with fixed 3′-ends and frequent 5′-truncations.

Amongst plants, the *Arabidopsis* mobilome is particularly rich at the species level. In his talk, **Leandro Quadrana** (IBENS, Ecole Normale Supérieure, Paris, France) indicated that the composition and activity of the mobilome varies greatly between accession numbers in the public databases. Natural variation of the mobilome is associated with sequence variants, notably in the MET2a gene, which encodes a poorly characterized DNA methyltransferase. TE mobilization is also governed by environmental factors. Accordingly, temperature annual range is a clear contributor to the variation in the LTR-retrotransposon ATCOPIA78 mobilization. L. Quadrana concluded his talk by showing that TE insertions have an important role in creating rare alleles with large effects on key adaptive traits (such as flowering time).

**Olivier Panaud** (LGDP, CNRS, Perpignan University, France) presented the results of a large-scale genomic survey of the retrotranspositional landscape of cultivated rice *Oryza sativa*, based on the analysis of the genomes of 3000 varieties. He showed that, as in the case of *A. thaliana*, a large part of the insertion polymorphisms are found in very low frequency, suggesting a strong retrotranspositional activity in rice germplasm and a high turn-over of TE-related sequences. Moreover, he presented some recent results on the assessment of transposition *in planta* in rice, using next-generation sequencing technologies, showing the impact of the down-regulation of RdDm pathway on transposition activation.

The analysis of variation in plants has revealed that their genomes are characterized by high levels of structural variation, consisting of both smaller insertion/deletions, mostly due to recent insertions of TEs, and of larger insertion/deletions similar to the human Copy Number Variants (CNVs). **Michele Morgante** (Universita di Udine, Udine, Italy) described the sequencing to high coverage of more than 100 grapevine entries in the public databases. He used a variety of approaches to detect structural variants of different size and origin, including *de novo* assembly of a selected set of genotypes, and proposed different mechanisms that could have generated and maintained the dispensable portion of the genomes and the genetic and epigenetic effects of the structural variants caused by TE movement.

TE repression in *Drosophila* gonads is dependent on the PIWI‐interacting RNAs (piRNA) pathway, where Argonaute proteins from the PIWI clade act together with piRNAs to target and silence active elements. **Abdou Akkouche** (IGH, CNRS, Montpellier, France) reported that in the adult female, Piwi is needed at a specific stage of embryo development to license piRNA production in the germline. Repression by H3K9 methylation of adult piRNA source loci requires embryonic Piwi expression. Their data indicate that the identity of piRNA source loci is then maintained in a Piwi-independent manner.

A hallmark of active centromeres is the presence of the histone H3 variant CenH3 in the centromeric chromatin. This ensures faithful genome distribution at each cell division. Using CenH3 as a centromeric marker, **Sandra Duharcourt** (IJM, CNRS, Paris Diderot University, Paris, France) discovered that in *P. tetraurelia*, centromeres are active in the germline micronuclei and inactive in the somatic macronucleus, although both nuclei originate from the same zygotic nucleus. Developmentally programmed centromere loss is caused by the elimination of DNA sequences associated with CenH3. The Duharcourt laboratory has set up a flow cytometry-based method, validated by imaging flow cytometry and Illumina paired-end sequencing, allowing isolation of pure germline nuclei for the first time. This innovative approach has made it possible to sequence, assemble and annotate the germline genome of *P. tetraurelia,* in particular transposable elements.

The distribution of Alu, L1 and SVA mobile elements throughout various primate genomes makes them useful tools for resolving population genetic relationships and primate phylogenetic relationships. These elements belong to discrete subfamilies that can be differentiated from one another by diagnostic nucleotide substitutions. In the last talk of the meeting, **Mark Batzer** (Louisiana State University, Baton Rouge, USA) reported that differential Alu amplification occurred in primates with about 200-fold variation in rates of retrotransposition. He and his colleagues have identified a new SINE in marmosets that has amplified for a long time period, since the radiation of new world monkeys, to a copy number of just over 2200 elements. Their results also indicate that the majority of current Alu retrotransposition in humans is derived from Alu Y lineage elements with just over 40 currently active loci.

## Conclusion

This is one of the most important meeting in the world in the field of transposable elements; it takes place in France every 4 years in alternation with its sister conference in the United States. Participants divided equally between Europe and the rest of the world. One more time, it was a great success and for once the sun joined us the full time! The tradition of a large international meeting on Mobile DNA will continue in the USA. The Keystone Conference on Mobile Genetic Elements and Genome Plasticity will be held February 11–16, 2018 in Santa Fe, New Mexico.

## Abbreviations

Bp: Base pair
ChIP-seq: Chromatin immunoprecipitation followed by massive DNA sequencing
CNV: Copy Number Variant
ERV: Endogenous RetroViruses
EVE: Endogenous Viral Elements
HERV: Human Endogenous RetroVirus
IES: Internal Eliminated Sequences
IFN: Interferon
Kb: Kilobase pair
KRAB-ZFP: KRAB-containing zinc finger protein
L1: LINE1
LTR: Long terminal repeat
NPC: Nuclear pore complex
ORF: Open reading frame
PFV: Prototype foamy virus
piRNA: Piwi-interacting RNA
PLE: Penelope-like element
PTS: piRNA-trigger sequence
RC-seq: Retrotransposon capture sequencing
RdDM: RNA-directed DNA methylation
RNA Pol III: RNA polymerase III
RNAi: RNA interference
RNP: Ribonucleoprotein particle
scnRNA: short non-coding scan RNAs
sRNA: small RNA
TE: Transposable element

